# Perceptions of rewards among volunteer caregivers of people living with AIDS working in faith-based organizations in South Africa: a qualitative study

**DOI:** 10.1186/1758-2652-13-22

**Published:** 2010-06-14

**Authors:** Olagoke Akintola

**Affiliations:** 1 School of Psychology, University of Kwa-Zulu Natal, Durban, South Africa

## Abstract

**Background:**

Volunteer caregivers are a critical source of support for the majority of people living with HIV and AIDS in southern Africa, which has extremely high HIV/AIDS prevalence rates. While studies have shown that volunteer caregiving is associated with negative health and socio-economic outcomes, little is known about the positive experiences of volunteers in the home-based care context in South Africa. The purpose of this study is to explore the perception of rewards among volunteers working in home-based care settings.

**Methods:**

This study uses a qualitative design. Qualitative interviews were conducted with a purposively selected sample of 55 volunteer caregivers using an interview schedule containing open-ended questions.

**Results:**

Volunteer caregivers derived intrinsic rewards related to self-growth and personal (emotional and psychological) development on the job; they also derived satisfaction from community members taking a liking for them and expressing a need for their services. Volunteers felt gratified by the improvements in their health behaviours, which were a direct consequence of the experiences of caring for terminally ill patients with AIDS. Extrinsic rewards came from appreciation and recognition shown by patients and community members. Extrinsic rewards also accrued to volunteers when the services they rendered made their patients happy. Perhaps the greatest sources of extrinsic rewards are skills and competencies acquired from training and experience while caring for their patients, and volunteers' ability to make a difference in the community.

**Conclusions:**

Insights into volunteer caregiver rewards provide opportunities for policy makers and programme managers to develop a model of home-based care that facilitates the accrual of rewards to volunteers alongside volunteers' traditional duties of patient care. Programme managers could employ these insights in recruiting and assisting volunteers to identify and reflect on rewards in the caregiving situation as a means of reducing the burden of care and sustaining volunteer interest in caregiving.

## Background

Informal caregivers are a critical source of support for the majority of people living with HIV/AIDS worldwide [1-4]. This is particularly true in the southern Africa region, which has countries with some of the highest HIV/AIDS prevalence rates in the world.

South Africa is severely affected by the epidemic; the Joint United Nations Programme on HIV/AIDS (UNAIDS) estimates that 18.1% of the adult population in the country were living with HIV/AIDS by the end of 2007 [5]. This makes South Africa the country with the highest number of people with HIV/AIDS in the world. The high proportion of infected people seeking health care in the public health system, coupled with HIV/AIDS deaths, as well as emigration of increasing numbers of health personnel to developed countries [6,7], has put the public health care system under severe strain.

Government is supporting home-based care as a way of reducing the pressure on public health facilities. This entails reduction in the length of patient stay in hospitals [4] through non-admission or early discharge of HIV/AIDS patients to homes to be cared for by family members. In reality, however, most family caregivers are ill-equipped and under-resourced to provide the needed care [4].

As a result, volunteers (hereafter "volunteer caregivers") are recruited from AIDS-affected communities and trained by non-governmental home-based care organizations, many of which are faith-based organizations [4,8]. They provide informal care and support for the increasing numbers of patients and their family caregivers across the country [4,8,9]. Volunteer caregivers therefore play a major role in the provision of informal care and are the only source of support for many affected families [4,8], but many of them are neither remunerated nor receive any pecuniary reward [4,10]. Although faith-based organizations constitute a large proportion of care organizations in South Africa, estimates of their proportion or the extent of their involvement in home-based care are unavailable.

Literature on family caregiving for people living with HIV/AIDS (PLHIV) worldwide has tended to focus on the negative implications of caring for the caregiver, usually referred to as the burden of care. For instance, family caregiving has been shown to result in poor health and socio-economic outcomes for caregivers [11-14]. In Ghana, Mwinituo [15] found that family caregivers of PLHIV experience stigma and discrimination and are isolated from sources of support.

Tarimo's study in Tanzania [16] reveals that caregivers, predominantly women, experience severe economic burdens from caring. Caring is reported to undermine the ability of women to work on the farm, thereby creating food insecurity. However, these studies present an incomplete picture of caregiver experiences as they focus mainly on the negative aspects of caregiving, ignoring the positive aspects.

Research in other informal caregiving contexts, namely those caring for the elderly, mentally ill, chronically ill and Alzheimer's patients, indicate that in addition to stresses, caregivers report positive perceptions (hereafter "rewards") as part of the caregiving experience [17,18]. For the purpose of this study, perceived reward is defined as the positive subjective feelings or objective changes, both internal and external, in the volunteer caregivers' lives resulting from their caregiving situation [17]. They include perceived positive feelings or obligations, pleasures, satisfactions, gratifications and positive outcomes from enduring a burden [19].

Schwartz's study among parents caring for their adult children with mental illness and chronic disabilities reveals that parents experience positive outcomes from fulfilling their duties and from learning about themselves [18]. Similarly, research among family carers of people with intellectual disabilities shows that although family carers feel that caregiving is stressful, they nonetheless find it satisfying and rewarding [20]. Indeed, it has been suggested that caregiver rewards could be a coping resource, serving as a buffer between caregiver burden and negative health consequences [21].

One of the few studies focusing primarily on the benefits of caregiving conducted among family caregivers reveals that individuals find meaning in providing AIDS care to family members and friends [22]. Given the experience of rewards among family caregivers in other contexts, as well as among family caregivers of PLHIV, it seems logical to surmise that volunteer caregivers of people living with AIDS would experience some form of rewards as well. Yet, little is to be found on volunteer caregiver rewards in the AIDS care literature

Much of the scant literature on volunteer caregivers in the HIV/AIDS context focuses on the experiences and stresses of volunteering [3,7,23-26], obscuring possible rewards and benefits. A UNAIDS study [25] found that stress is pervasive among AIDS care volunteers and that this emanates from the nature and context of care work. Akintola's study [7] shows that volunteers caring for PLHIV confront a myriad of challenges, including the sheer difficulty of caring, and maintaining confidentiality among others.

However, research among volunteers working in AIDS service organizations in Ontario, Canada, reveals that volunteers experience intrinsic rewards, such as improved self-esteem and health, and self-actualization, as well as extrinsic rewards, such as recognition, constructive feedback and participation in decision making [27]. However, little is known about rewards among volunteer caregivers of people living with AIDS in the home-based care context in Africa.

Therefore the aim of this study was to explore volunteer caregivers' perceptions of rewards of providing care to people living with AIDS. The findings presented in this article are a part of a larger study that explored the role and lived experiences of volunteers in home-based care. The results relating to volunteer caregiver burdens are presented elsewhere [7]. Given the negative experiences associated with volunteering in AIDS care, research on the rewards derived by volunteers in this setting could provide insight into the critical roles that rewards could play in reducing the burden of care on volunteer caregivers, as well as factors sustaining volunteer interest in community-based care.

The choice and social exchange theory posits that interpersonal relationships may be sources of rewards, including feelings of gratification, status, social approval, autonomy and financial gain [28,29]. It suggests that people will use strategies to avoid costly interactions and seek rewarding social statuses, relationships and feeling states. The theory emphasizes the interdependence in dyadic relationships and mutual exchanges proposing that, within dyadic relationships, the behaviours of each participant influence the costs and rewards of the other. According to the theory, people will attempt to minimize situational losses so that their perceived costs do not greatly exceed their perceived rewards. Put differently, people try to make the best of every difficult situation.

The choice and exchange theory has been used in previous studies examining rewards among family caregivers in other contexts [17,20]. This theory, though not previously applied to volunteer caregivers of people with AIDS, might hold the promise for illuminating rewards among volunteer caregivers. In this regard, there is a need to examine the behaviour of and interaction between volunteers and their patients, as well as the caregiving situation and how these might influence volunteer caregivers' perception of rewards in the caregiving process.

## Methods

### Study setting and context

The study was conducted in six Zulu-speaking communities, made up of five semi-rural communities (townships) and an informal settlement, all located on the outskirts of Durban in the KwaZulu-Natal province of South Africa. The communities were selected with the help of care organizations. The care organizations were selected based on the following criteria: (1) willingness to facilitate easy access to their volunteers; (2) a large volunteer base that guarantees a large pool of potential participants; and (3) provision of services to two or more communities.

The two care organizations that met these criteria are faith-based organizations (FBOs) and were recruited for the study, given that FBOs play a critical role in home-based care in South Africa [4,8]. The two FBOs use a similar curriculum to train volunteers from HIV/AIDS-affected communities to provide care and support for patients in their homes. They work in 16 communities and have some of the highest number of volunteers (550 individuals combined) of all care organizations in the Durban metropolis.

The organizations were started on the Christian philosophy of reaching out to and helping others, and they seek to recruit volunteers from AIDS-affected communities. However, they do not restrict the recruitment of volunteers to people of the Christian faith, but extend it to all community members who indicate willingness to volunteer in AIDS care. There seems to be a tradition of helping others in these communities, which has been variously referred to as *Ubuntu*, an African humanist philosophy that emphasizes the interconnectedness and mutual dependence of individuals and the need for people to help each other. Therefore, there seems to be a convergence in the philosophies of the organizations, as well as those of the communities that they serve.

The FBOs operate a similar model that recruits volunteers from HIV/AIDS-affected communities and trains them to provide care to the large numbers of people desperately in need of care. They operate an office with a small paid staff complement that includes nurses, administration staffers, drivers and community coordinators.

Volunteers are trained for a six-week period on the theory and practice of home-based care, counselling, tuberculosis prevention and treatment, and nutrition. Thereafter, volunteers visit the HIV/AIDS-affected homes to provide the necessary care to patients and support to their families. Volunteers provide basic nursing care to patients and assist with feeding, bathing, dressing and transferring patients. They also assist with housework, shopping, cooking collecting water, transportation to health facilities and making phone calls.

Volunteers also provide spiritual care and psychosocial support, such as assisting their patients and their families in securing government welfare grants. They also provide directly observed treatment, short course (DOTS) to tuberculosis patients, but none of the volunteers were involved in provision of antiretroviral (ART) treatment as ART was not provided to patients by either of the FBOs at the time of the study. Supervision of the volunteers is carried out by community coordinators and facilitators.

The organizations' inability to secure funding for volunteer stipends makes it impossible for them to provide these or other forms of remuneration to volunteers. However, the organizations operate a progressive and developmental model that provides opportunities for capacity and career development for their volunteers within the limits of the available funding. In addition, the FBOs provide incentives, such as: provision of groceries; end-of-year gift vouchers; food parcels for the very needy; umbrellas; shoes; allowances for uniforms and subsidies for their children's school fees; toiletries, including sanitary towels; and in the case of one FBO, access to free health care in the affiliate hospital, including antiretroviral (ART) treatment. In addition to the six-week training provided, one of the FBOs enrols volunteers, who excel at their work, in a palliative care training course in its affiliate hospital and pays them allowances for the six-month period of their training. These incentives help sustain volunteer interest in their work.

### Participants

Following Ulin *et al*'s [30] suggestion that qualitative researchers select information-rich cases that are representative of a range of experiences, a purposive sample was recruited. We sought to: (1) include participants with a range of experiences and perspectives from several communities; and (2) collect data in order to reach saturation in each of the study communities. To achieve this, we planned to interview at least 10 volunteers with a range of socio-demographic characteristics in each of the study communities. However, we were only able to interview and reach saturation with at least eight participants in six of the 16 communities, making a total of 55 volunteers (see Table 1).

**Table 1:** Number of participants recruited from the six study communities

Communities	Number of participants
Community A	8
Community B	11
Community C	11
Community D	9
Community E	8
Community F	8

Total	55

Participants were recruited if they were: enrolled and trained as volunteer caregivers by one of the FBOs; not receiving remuneration; providing care to someone with clinical AIDS for a minimum of three months; and willing to participate in the study. Table 2 provides participants' socio-demographic information.

**Table 2:** Socio-demographic information of research participants

Items	Frequency
**Gender**	
Male	2
Female	53

**Age**	
19-24	5
25-50	45
51-55	5

**Highest level of education attained**	
Primary	12
Secondary	42
Tertiary	1

**Marital status**	
Single (never married)	45
Married	6
Divorced/widowed	4

**Religion**	
Shembe	3
Zionist	12
Apostolic	8
Methodist	3
Catholic	26
Presbyterian	3

**Employment status**	
Tailor	2
Trader	4
Hairdresser	1
Domestic worker	4
No employment	44

**HIV status**	
HIV positive	4
HIV status unknown	51

**Treatment status**	
On ART	4
Treatment status unknown	51

**Length of time in AIDS volunteer work**	
< 1 year	5
1-2 years	34
3-4 years	11
5-6 years	5

### Procedure

This qualitative study made use of open-ended interviews, and took place between December 2003 and April 2005. After the management of the non-governmental organizations was approached and agreed to participate in the study, they convened meetings with volunteers where the purpose of the study was discussed. Volunteers who consented to participate in the study worked with interviewers to agree on times and venues of the interviews. Interviews were conducted in community resource centres, church multi-purpose hall, councillor's offices or participants' homes, depending on participants' preferences and logistics. Following ethical guidelines, all participants were given information about the study and asked if they would still want to be interviewed. All participants gave verbal informed consent before interviews were conducted.

Interviews were conducted using interview schedules consisting of open-ended questions. The interview schedules focused on socio-demographic variables and a range of questions around their roles and lived experiences as volunteers and perceptions about their volunteering work. All interviews were conducted in the local language, isiZulu, by three trained interviewers and each took between 40 and 120 minutes to complete.

The study protocol was approved by the boards of the care organizations and the ethical review board of the Faculty of Human Sciences of the University of KwaZulu-Natal, South Africa.

### Data analysis

All interviews were audio-taped, and data was transcribed and translated independently by two research assistants. The separate transcripts from each of the translators were thereafter compared, and discrepancies were discussed and clarified in order to ensure high-quality data. Analysis began with immersion in the data and was guided by the literature on informal caregiver rewards and that on rewards of AIDS volunteers. Using constant comparison [31], data was examined for text relating to perceived benefits, satisfaction, gratification and positive feelings relating to volunteer care work. Themes were identified and coded, and thereafter classified into the two core reward themes (intrinsic and extrinsic) identified in the literature [27].

Pieces of text relating to each category were then grouped together and labelled accordingly. At the same time, data was constantly checked to identify new themes that may emerge [32]. In order to ensure that volunteers were not providing socially desirable responses, data was validated by discussing findings with some volunteers and volunteer coordinators and facilitators.

## Results

Caregiver rewards flowed from the caregiving situation through volunteers' interactions with patients and their families, as well as AIDS-affected community members. The interaction between the home-based care organizations, as well as the volunteers, also produced rewards. Drawing on the literature on volunteer and caregiver rewards discussed earlier, the findings are classified into two core themes/categories: intrinsic and extrinsic rewards.

### Intrinsic rewards

#### Learning virtues

Many of the volunteers indicated that volunteering enabled them to achieve self-growth and personal emotional and psychological development. This was a direct consequence of engaging on a daily basis with terminally ill people with AIDS, who have diverse care needs and are extremely demanding. These experiences led to the discovery or development of "the self". Volunteers viewed these rewards as virtues learnt while caring and were captured in phrases such as: "volunteering has made me a better person"; and "my life is better now than before".

Volunteers indicated that love and patience were the main virtues that they learnt from providing care to patients. Given that caregiving is a strenuous and demanding activity, volunteers learnt over time how to love and be patient with the ill, which was seen as a great personal reward. One said, "I've learnt that a sick person must get love unconditionally. I must not discriminate but treat them like my brother or sister." Another said, "[I've learnt] to be patient and to be patient for the sick person."

Patience was seen as a virtue that is obligatory for providing care. As one volunteer put it, "If you are not patient, you can't take care of a sick person." For yet another, self-growth came in form of lessons learnt on love and non-discrimination, and this led to a complete transformation of long-held attitudes and perceptions towards ill people and to life generally.

One participant, who could not stand ill people and dirt or filth prior to enrolling as a volunteer, said:

I grew up as a *nyanya *(who feels easily repulsed) person, but I learnt that I must not *nyanya *(be easily repulsed). I used to get repulsed easily before, but now I have learnt that I have to love the sick people. They have the same body parts as I do so I must not discriminate against them even though they are sick.

For some participants, self-growth came through learning how to show compassion. Volunteers gradually came to understand the need for a change in their self-centred attitude to that of selflessness:

I can say that my life has changed for the better because as a person, I grew up not worrying about another person's problem. And this (volunteer care work) has taught me to help other people, especially those that are sick and I have learnt to be concerned about other people's problems.

Some of the volunteers indicated that they acquired communication skills from the experience of dealing with difficult caring situations while providing care to the ill. "I have learnt to communicate with [the] community and everyone in general," one said.

Inner strength and confidence, another aspect of self-growth and psychological development, seemed to be a spin off of the skills and experience that volunteers acquire while providing care:

I have learnt so much in the sense that if I see a sick person I am the first person who tries to give him/her help and ask for their needs and the part of the body that is paining them.

#### Feeling liked and needed

Volunteers perceived that they were liked by their community members. One participant said, "The community likes me very much." Another felt gratified by the remarkable increase in the volunteers' clientele and perceived this as an expression of how much they were needed by the community, saying, "People are beginning to come in numbers asking for help from us." Yet another felt gratified by community members' affirmation of her competence:

Even now other volunteers who have terminally ill patients used to come and call me if they have problems with their patients because they know that I have a lot of experience

#### Health behaviour change

Engaging with the patients on a daily basis provided volunteers with the opportunity to change negative health behaviours. The most frequently mentioned change was in sexual behaviour. Care work enables volunteers to witness, first hand, the pains and sufferings of PLHIV, and this leads to introspection:

I am traumatised having seen how my patients suffer and I learnt a lesson that if I do not live my life responsibly, I might find myself in the same situation like she (the sick person) was, I saw the symptoms of AIDS and I have learnt to take care of myself, eat proper nutritious food and to avoid unsafe sex.

I learnt that I should take good care of my life. So...I do not sleep around without using protection for sex.

The knowledge and skills acquired by volunteers on hygiene and nutrition motivates them to change their eating behaviour:

We are being taught the way of eating and how to prepare nutritious food and then I always go back and try it at home.

### Extrinsic rewards

#### Enjoying appreciation and recognition

An important reward for volunteering is the appreciation shown by patients and their family members, as well as recognition shown by community members and the care organization. Appreciation and recognition was a recurrent theme in the narratives of volunteers, underscoring the importance that they attached to it:

There is nothing as inspiring as seeing people appreciating our services and even some will buy us some presents though we're not allowed to take gifts from patients.

Community members were initially reluctant to allow volunteers to access ill family members because of fear of stigma. This made volunteering work difficult and frustrating. However, after a lot of hard work and community education, community members were gradually coming to terms with their work, and that led to a softening of the community stance. Volunteers viewed being allowed access to patients as a form of reward:

The community has come to a stage of accepting us as helping them, and people are very cooperative with us. They knock at our doors and [refer] us to other people who are sick and now I have a lot of people under my care. And God is helping us a lot in dealing with the problems that our community is facing.

#### Giving pleasure to patients and community members

Another source of extrinsic reward is the pleasure that volunteers derive from making their patients and their family members happy. Participants' presence in the communities improves patients' access to care, thereby making their patients and family members happy. In this case, the volunteers' gratifications are inextricably linked to the happiness felt by the people that they serve:

All our neighbours feel very happy because we are there for them always.

The community is very happy about us because they are the ones who are referring other patients to us.

#### Acquiring skills and competencies

Volunteers saw their training and day-to-day, hands-on experience while providing care to a number of ill people as a rare opportunity to learn skills. The fact that they had indeed gained confidence to care for their ill family and community members was a particular source of tremendous gratification for volunteers:

This volunteering thing has helped me a lot because of AIDS...I didn't know anything about AIDS, but now I can help people with the knowledge I get from training and the daily experience.

What I have gained, which I am happy about, is the training. I did not know anything about it before but now I know. I also learnt about DOTS.

An additional benefit of the training received and hands-on experience was increased sensitivity to the plight and rights of the ill:

I always learn a lesson from here (volunteer care work). For instance, I see people who do not take care of their sick members and therefore I learn that if that happens in my family I am going to be seriously opposed to it because I know how sick people feel.

Another volunteer expressed satisfaction that the training she received had put her in a better position compared with that of her peers who were not trained:

Myself, I'm satisfied...I am very much [more] privileged than those who did not get the training.

#### Personal effectiveness

Volunteers found great reward and satisfaction in the positive health and socio-economic outcomes derived by patients as a consequence of their services. A common thread in their narratives was that of "making a difference" in the lives of the patients and community:

Now that some of my patients got their pensions, they buy food and I am feeling happy and I am encouraging them to buy things for the house. Many people are now beginning to see the difference we are making in the community.

I have got the knowledge on how to treat people who are suffering from AIDS. There are patients that I found...lying on the bed and after I came there, they can walk, they can talk and they have got their (government-awarded social) grants and that is what makes me proud of my work because I can see the difference I am making.

At the same time, a spin off of this is that it engenders community trust, which was seen as a reward by volunteers:

Myself, I have come to be known and trusted by so many people and I like that because it shows that I'm making a difference in the lives of other people.

Volunteers' ability to facilitate patients' disclosure of their HIV status to family members was seen as a great reward:

You find that if the sick person has not disclosed the HIV status to the family, they will just call us as volunteers and they tell us about the status and they will ask us to tell their family about that on their behalf. We are making things easy for the patients to approach life very positively.

## Discussion

Previous studies on family and volunteer caregiving for PLHIV have focused on the burden of caregiving, such as adverse physical, mental and socio-economic consequences [7,33,34]. Drawing on the choice and social exchange theory, this study provides insight into rewards derived by volunteers providing care to people living with AIDS in their communities. It reveals that volunteers also experience rewards from caregiving, in addition to the negative outcomes that have dominated the literature on family [14-16] and volunteer [7,25] AIDS caregiving.

While few studies have documented the existence of rewards among family caregivers of PLHIV and volunteers working in AIDS service organizations [22,27,35], there is no known published study to date on rewards among volunteer caregivers in home-based care, particularly in the African context.

The choice and social exchange theory helps elucidate the rewards that volunteer caregivers receive as a part of the caregiving experience. In this study, volunteers report experiencing a number of intrinsic and extrinsic rewards. Most of these rewards seem to proceed out of their sustained engagement with caregiving. Therefore, many of the volunteers find rewards in the course of doing their volunteer work, providing care to people living with AIDS.

This, in itself, is instructive, for it suggests that people can also derive positive benefits alongside the negative experiences documented among family and volunteer AIDS caregivers [7,15,34]. This is in accord with the choice and social exchange theory that posits that people attempt to minimize their losses so that perceived costs do not greatly exceed perceived rewards [28]. Lazarus and Folkman [36] argue that the appraisal of a potentially stressful situation is central to how the situation is perceived by an individual. The authors also suggest that individuals need to find something meaningful when enduring difficult circumstances.

While it is possible that volunteers experience rewards as a normal process in caregiving, it could also be that volunteers carry out secondary appraisal of the difficult caregiving situation, and thereafter identify and draw on the positive experiences accruing from their work to cope with the challenges [7]. In other words, rewards could be used as a coping resource [21,36].

As Grant and colleagues [20] argue, intrinsic rewards can indeed serve as coping resources. Lawton *et al *[21] also suggest that caregiver rewards might be used to buffer the detrimental consequences of caregiving. Further, Berg-Weger *et al *[37] have shown that there is a link between positive perceptions about caregiving and improved caregiver wellbeing. One could therefore surmise that if volunteer caregivers are able to perceive their work in a positive light, they might experience better wellbeing.

Given the potential role of perceived rewards in buffering caregiver burdens, home-based care project managers, as well as primary health care practitioners, such as mental health care nurses and counsellors, could help volunteers identify and reflect on the positive rewards that volunteers experience in the caregiving process. This could potentially be used to reduce the negative consequences of informal AIDS care.

Studies have shown that lack of community appreciation and hostility of community members to volunteers are key stressors for volunteers [7]. However, volunteers in this study find appreciation of their services by the community members inspiring and satisfying. This echoes the findings by Carlisle [22] among family caregivers and Crook and colleagues [27] among volunteers working in AIDS care organizations. Their studies show that AIDS care workers experience a sense of pride and satisfaction when provided with positive feedback and recognition. The finding points to the need for communities to be enlightened and educated on the value of treating volunteers decently and warmly, and of showing appreciation and recognition to volunteers.

Of all the rewards mentioned by volunteers, acquisition of skills and expertise, as well as the manner in which volunteers perceived that they were effective in their work, seem to be the most important to them. This highlights the need for managers to emphasize the value and/or benefits of the training and expertise that volunteers receive. While making certain that volunteers have sufficient opportunity to discuss their negative experiences, managers could help them focus and reflect on their success stories as well.

Additionally, care policies and programmes should be geared towards producing an enabling environment that will facilitate the accrual of these rewards to volunteers by providing ongoing training to volunteers and provision of material, technical and financial resources necessary to make volunteers' work more effective and worthwhile.

Volunteers find satisfaction from helping patients disclose their status. This highlights a major area of impact for volunteers given the stigma and discrimination surrounding HIV and AIDS and the unwillingness of HIV-positive individuals to disclose to their family members [7,24]. Previous studies have shown that volunteers carry the burden of non-disclosure because patients disclose to volunteers with firm warnings not to disclose to their families [7,33].

At the same time, studies also show that home-based care programmes can facilitate acceptance of HIV status and disclosure by those who are living with HIV and AIDS, both within and beyond their family network and households [38,39]. However, because these studies make use of quantitative designs, they do not provide insight into how care programmes facilitate disclosure. Since some volunteers find it gratifying to be able to facilitate disclosure among HIV-positive people, it appears that they possess skills which help facilitate the disclosure process.

This highlights the need for more skills training that will enable many more volunteers to continue to play this role effectively. In this regard, volunteers can serve as intermediaries between family members and the patients, and they need to be given more training on how to counsel patients on disclosure and handle confidential information professionally while avoiding detrimental effects on their mental health and social wellbeing [7].

Of critical importance is the finding that volunteering facilitates reflection about the volunteers' need to change their health behaviour, particularly sexual behaviour. This is consistent with, and may be explained in the light of, the health belief model developed by Rosenstock [40]. Rosenstock posits that the knowledge of someone close who is infected with HIV could serve as "cue to action" or motivation for changing sexual behaviour. According to Rosenstock, cues trigger appropriate health behaviour.

According to Mack [41], working with PLHIV challenges the care provider's denial of his or her own risk of HIV, as well as risks of friends, families and children. Thus, Mack argues that caring for people with AIDS can shatter the denial that separates the caregiver from the care recipient. In light of these findings, there is need for research on the protective effect of caregiving on sexual risk behaviour, i.e., research is needed to explore the impact of caregiving on sexual risk behaviour.

While the study provides insight into volunteer caregiver rewards, a number of limitations must be acknowledged. First, the use of a qualitative design precludes quantification of the proportion of volunteers experiencing rewards and the most important rewards to volunteers. It also prevents generalization of findings.

Second, the sample was drawn from two faith-based organizations that do not provide remuneration. Rewards experienced by volunteers working in other settings, in other provinces or those working with organizations providing remuneration might differ.

## Conclusions

This study takes a departure from previous studies on AIDS caregiving, which have focused predominantly on caregiver burdens, and provides insight into volunteer caregiver rewards. The study shows that volunteer caregivers receive different kinds of rewards as part of their caregiving experiences, which may be used to reduce the effect of the burden of care on volunteers.

However, the effects of these rewards may not counterbalance the effects of the burden of AIDS caregiving: although the rewards may serve as a buffer between caregiver burden and negative outcomes, they may not necessarily obliterate the burden that volunteer caregivers experience. Rewards might, however, help reduce subjective, as well as objective, burden among volunteer caregivers. This may enable volunteers to continue their work cognisant that certain rewards are accruing or will continue to accrue to them. Rewards therefore might play a major role in sustaining volunteer interest in working with care organizations. This appears to be welcome news for care organizations struggling to keep volunteers in the absence of funds for remuneration.

Given the capacity problems in the public health care system and the need to scale up antiretroviral therapy for PLHIV, government is likely to continue to rely on volunteers to play a major role in AIDS care and treatment for a long time to come. Therefore, policy makers and funding agencies could help care organizations build home-based care management models that take cognisance of the value that volunteers attach to receiving objective and subjective caregiver rewards in both the recruitment and management of volunteers.

Such a model of home-based care should have a dual focus, facilitating the accrual of rewards to volunteers and at the same time providing an enabling environment for volunteers' traditional duties of patient care. In this regard, care organizations could form partnerships with AIDS-affected communities and funding agencies to facilitate working environments that enable the accrual of various kinds of rewards to volunteers.

For example, care organizations could prioritize skills training and the provision of adequate resources that help volunteers make a difference in the lives of their patients and their families as part of their core mission. This should not be only for the benefit of the patients, but also to serve as a reward for volunteers. Further, volunteers should also be encouraged to identify and discuss psychological and emotional, as well as physical, rewards in caregiving during feedback and counselling sessions, informal discussions, reviews and appraisals of volunteers.

At the same time, there is need for caution so that the burden of care is not overlooked or relegated to the background. Therefore, studies that explore and quantify the role of rewards in moderating volunteer caregiver burden are urgently needed.

## Competing interests

The author declares that they have no competing interests.

## Authors' contributions

OA was responsible for conceptualizing and designing this study. OA was also responsible for conducting, analyzing, and writing up of drafts and the final version of the manuscript.
